# Collectanea Medica: Consisting of Anecdotes, Facts, Extracts, Illustrations, &c.

**Published:** 1824-09

**Authors:** 


### COLLECTANEA MEDICA:

CONSISTING OF

### ANECDOTES, FACTS, EXTRACTS, ILLUSTRATIONS, &c.

*Relating to the History or the Art of Medicine, and the Collateral Sciences.*

Floriferis, ut apes, in saltibus omnia libant,
Omnia nos, itidem, depascimur aurea dicta.

---

ART. I.—*Case of Laryngeal Inflammation,* by JOHN CRAMPTON, M.D. *where Tracheotomy was successfully performed by* RICHARD CARMICHAEL, Esq. M.R.I.A. one of the Surgeons to the Richmond Surgical Hospital, &c. &c.—(Transactions of the Association of Fellows and Licentiates of the King and Queen's College of Physicians in Ireland, Vol. IV.)

THE following case of laryngeal inflammation is offered to the Association, principally on account of the successful result of the operation of tracheotomy. It may be considered as an instance of acute laryngitis supervening on a chronic tracheal and bronchial affection. The operation has often failed, from the disease extending down to the different tissues of the lungs. In the present case, the recovery of the patient appears to be fairly attributable to the large opening, which not only allowed free inspiration and expiration, but permitted the mucous and other collections to be expelled without any difficulty or interruption. The event of this case, then, encourages us not to despond of the issue of tracheotomy, even when the disease has extended down the trachea into the lungs, and where from ordinary treatment we have nothing to hope.

Maria Dunne, æt. thirty, a strong woman, was admitted in the Whitworth Hospital, on the evening of the 25th October, 1823. Until the latter end of August, her health had been perfectly good: at that period, after exposure to wet, she was affected with a troublesome cough, accompanied with copious, tough expectoration. On being further exposed to cold, her cough became increased, attended with hoarseness and sore-throat. On the 21st October, she was first seized with oppression, difficulty in breathing, and a dull pain from the region of the larynx down along the course of the sternum. For these symptoms no appropriate remedies were resorted to: on the contrary, her friends advised spirituous liquors. A remedy so congenial to her feelings she indulged in freely for three days antecedent to her admission into hospital; after this excess, the symptoms were much aggravated. On the evening of her admission, her cough was frequent, with copious but tough expectoration; her breathing difficult, attended with a hoarse, brazen sound; her pulse accelerated. The usual purgative remedies were directed. Towards night, however, her respiration became so much obstructed, her countenance appearing anxious, pulse 100, whilst she experienced great difficulty in getting up the tracheal mucus, that twelve ounces of blood were taken from the arm, and an expectorating mixture directed for her. She experienced immediate relief from the bleeding, soon after which she fell asleep, and passed a tranquil night.

26th.—The dyspnœa had returned. At the time of my visit, her chief complaint was pain in the region of the larynx; she pointed also to the scrobiculus cordis, as a place where she suffered considerable distress: pulse 112. A repetition of the bleeding was directed, also pills with ipecacuan and calomel every four hours, with a nitrous mixture. An emetic was ordered for evening. These remedies were all practised, but she had experienced no relief at eight o'clock in the evening.

27th.—In the morning there appeared to be an evident exacerbation of all the symptoms: the cough is increased in frequency, its sound is like croup; respiration difficult, and obstructed by a quantity of viscid mucus; pulse 120; pain in the region of the larynx and under the sternum, below the cartilagio ensiformis, distressing; she is, however, pale and exhausted. It was not judged expedient at this juncture to take blood from the arm, the distress appearing to be limited to a circumscribed space; but six leeches were directed to the region of the larynx, an emetic of ipecacuan, afterwards a continuance of the pills, with calomel and ipecacuan every four hours, besides the nitrous mixture. She was somewhat relieved after the leeches and the emetic; her pulse in the evening 126.

28th.—A very distressing night. At six in the morning, her pulse was very rapid and small; her countenance showed great anxiety; both inspiration and expiration required considerable exertion, and were accompanied with a wheezing noise; her bowels were free. Eight leeches were then applied to the exterior of the larynx, the other treatment being continued. From the report at noon, it appeared that she felt some temporary alleviation after the leeches: her countenance, however, was quite livid, and expressive of extreme anxiety, with a look of wild despair; her eyes protruded; respiration was nearly impracticable; the extremities, as well as the whole surface, were becoming cold; pulse fluttering and feeble, above 130. She was, however, in perfect possession of her intellect, and implored that something might be done to afford her relief under the extreme urgency of her suffering.

It was now evident that a termination to her existence was at hand, unless the organs of respiration were restored to a performance of their functions. The rima glottidis was, I presumed, nearly closed, and the quantity of viscid creamy mucus almost completed the obstruction. The conclusion I formed at this juncture was, that tracheotomy alone afforded any chance of saving the life of this patient. I directed, however, a continuance of the calomel and ipecacuan pills, and a cordial mixture. I requested also that Mr. Carmichael, on whom the surgical duty of this hospital devolved, should be immediately sent for, and that the operation, if it met his approbation, might be performed.

Mr. Carmichael soon arrived, and approving of my suggestion, immediately performed the operation of tracheotomy. On the opening being freely made, she experienced immediate relief. The irritation of the trachea, for a time, produced troublesome fits of coughing; after which, considerable quantities of purulent-looking mucus were expelled the wound; the colour of her countenance was soon improved, and the temperature on the surface and extremities shortly became restored, without any additional remedial effort. Towards evening, having expelled much gross mucus through the wound, and the irritation in the trachea subsiding, she became composed: the frequency of the cough, however, prevented her from sleeping.—The same remedies were continued.

29th.—Is much better to-day: pulse 124, stronger and more steady; no longer feels the pain in the larynx or in the scrobiculus cordis;* even when the wound is stopped for a time, she speaks with more facility than previous to the operation. She breathes freely, and expectorates through the aperture; but, if the orifice is obstructed for any time, she feels great distress, and expresses considerable alarm at the idea of its closing. Cough is less frequent; she expels some mucus from the mouth.—In addition to her other remedies, an anodyne antimonial draught was directed at night.

30th.—She slept the greater part of the night without the opiate. Her voice, notwithstanding the wound is still open, has returned; the distress in the region of the larynx is greatly diminished; expels now more mucus by the mouth than by the tracheal aperture; pulse 120; countenance has resumed its natural appearance.—An expectorating mixture was directed to-day, (the calomel and ipecacuan being still continued,) and an aperient draught, as her bowels were slow.

31st.—Has slept a good deal. Cough abates, except after a long sleep, when the mucus collects; the irritation of this disturbs her: after coughing and expelling the greater part of the accumulation through the wound, she again falls asleep; pulse 116.—Medicines continued; beef-tea.

November 1st.—Still improves; breathes easy through the wound; pulse 120.—Calomel and ipecacuan omitted. To take the expectorating mixture and pills, with assafœtida at night.

2d.—Is to-day slightly feverish and flushed; pulse 120; edges of the wound are much inflamed.—Eight ounces of blood were taken from the arm; mixture with acetated ammonia and a purgative directed, the other medicines being omitted, and the low diet resumed.

3d.—Became cool shortly after the bleeding; pulse 98; cough less troublesome; wound little more healthy.—A cough mixture, with tincture of opium.

4th.—Slept well, breathing at times only through the mouth, unaccompanied by any hissing noise; pulse 90; appetite good.

5th.—Progressive in improvement; breathes naturally through the larynx. Wound allowed to close.

11th.—Her convalescence complete. Wound now quite closed, and begins to cicatrize. Respiration, as well as all the other functions, are performed naturally.

She was discharged, perfectly cured, from the hospital on the 22d of November, being detained longer than was necessary, on account of the

* This distress was probably in the heart, for want of its accustomed stimulus of renovated arterial blood.

peculiar interest of the case. In collecting the notes of this case, I have had much assistance from Mr. Lewry, the clinical clerk of the hospital. To his close attention, also, to this patient, next to the promptness with which the operation was performed, I am disposed, in a great measure, to attribute her recovery.

### Mr. Carmichael's *Account of the Operation.*

The history and symptoms of Maria Dunne's case have been so accurately detailed by Dr. Crampton, that it is unnecessary for me to occupy the time of the Association, except by a simple detail of the steps of the operation of tracheotomy, which was performed a few minutes after I first saw the patient in the afternoon of the 28th October, 1823. Indeed, the difficulty of breathing was so great, that it was obvious there was but little time to spare; and my only doubt respecting the propriety of the operation arose from the apprehension that it was now too late to afford her even this chance of prolonging life, as I feared, from the rapidity of her pulse and appearance of her countenance, that effusion had already taken place into the lungs. However, the fortunate result of the measure tends to evince that, even in cases apparently extreme, success may follow its adoption.

With the assistance of the resident pupils of the Richmond Hospital, Messrs. Belton and Bushe, and Mr. Lewry, Dr. Crampton's clinical clerk, I proceeded to perform the operation; having placed the patient on a chair opposite a window which afforded a good light, her head being thrown back, and firmly supported by an assistant behind.

Having ascertained the lower edge of the thyroid gland, an incision, about an inch and a half in length, was made through the integuments from this point to within a finger's breadth of the sternum. The incision was continued between the sterno-hyoidei and the sterno-thyroidei muscles, until the rings of the trachea were fairly exposed. This momentous part of the operation was performed in a few seconds; for, with the aid of my assistants, who separated the lips of the wound by means of retractors, I was enabled to see the parts which it was necessary to divide; and, by the fore-finger of the left hand, I successively felt each part previous to the application of the knife, the edge of which, after the division of the skin, was directed constantly upwards during the subsequent parts of the operation; and these precautions were a sufficient security against wounding the arteria innominata, which sometimes rises above the sternum, or from dividing any known or anomalous arterial branch of magnitude which might course in front of the trachea.

This was all happily accomplished, with very little delay, and the loss of a very few drops of blood, notwithstanding the difficulty which the perpetual motion of the trachea opposes in a person incessantly gasping for breath. Two, or perhaps three, of the rings of the trachea were then divided from below upwards, and this opening was immediately widened by means of a large pair of sharp-pointed scissors, such as I employ for the operation of hare-lip. With these I had provided myself, knowing, from previous operations, the difficulty of removing by the knife a slip of the rings of the trachea, as recommended by Mr. Lawrence. Some little address is necessary in performing this part of the operation. The sharp point of one blade was introduced at the lowest part of the opening in the trachea, and directed outwards so far as to admit, on the closing of the blades, of the division of an extent of the tracheal rings, equal to one-half of the opening already made in them: the flap thus made was laid hold of by a pair of dissecting forceps; the point of the scissors was again introduced into the trachea, where the last incision terminated, and carried inwards towards the central opening. Similar incisions in the same manner were made on the opposite side, and a diamond-shaped opening, capable of admitting the point of the little finger with ease, was left in the trachea.

The patient coughed up a large quantity of thick viscid phlegm through the opening, and seemed so much relieved, that the appearance of suffocation, and excessive anxiety which her countenance displayed previous to the operation, immediately disappeared. I am satisfied that, if the operation had been confined in this, as well as in other instances which have occurred to me, to a mere division of the rings of the trachea, that no beneficial result could have followed the measure; and that even the introduction of a tube, which is objectionable on two accounts, would not be attended with any advantage: for, first, a tube excites intolerable irritation, with violent fits of coughing; and, secondly, the apertures in it cannot be sufficiently large to permit the expulsion of the viscid phlegm, which accumulates in most of those cases where tracheotomy is necessary.

It has been suggested to me by a friend, that a pair of curved scissors, such as are used for operations on the eyes, would be better adapted for removing a portion of the trachea by means of two elliptical incisions. I have no doubt but that this part of the operation would be more easily performed in this way, but I doubt whether they would make an opening sufficiently large for the passage of viscid mucus; and it cannot but be of advantage that practitioners should be acquainted with a certain mode of effectually performing this operation by means of a simple instrument, which is always at hand. The successful result in the present instance sufficiently proves that the removal of a small portion of the trachea is not followed by any permanent inconvenience.

The only surgical attention afterwards necessary, was the frequent removal of mucus from the aperture in the trachea, by means of a probe armed with lint. The orifice, however, became frequently clogged, notwithstanding its large size, during the first week after the operation. The mucus was occasionally cleared away by the attending pupil, and even sometimes, when the patient expressed much uneasiness, by the nurse-tender of the ward, and in some instances by the patient herself with a sponge.

